# ESKAPE Bacteria and Extended-Spectrum-β-Lactamase-Producing Escherichia coli Isolated from Wastewater and Process Water from German Poultry Slaughterhouses

**DOI:** 10.1128/AEM.02748-19

**Published:** 2020-04-01

**Authors:** Mykhailo Savin, Gabriele Bierbaum, Jens Andre Hammerl, Céline Heinemann, Marijo Parcina, Esther Sib, Alexander Voigt, Judith Kreyenschmidt

**Affiliations:** a Institute of Animal Sciences, University of Bonn, Bonn, Germany; b Institute for Medical Microbiology, Immunology and Parasitology, University Hospital Bonn, Bonn, Germany; c Department for Biological Safety, German Federal Institute for Risk Assessment, Berlin, Germany; d Institute for Hygiene and Public Health, University Hospital Bonn, Bonn, Germany; e Hochschule Geisenheim University, Department of Fresh Produce Logistics, Geisenheim, Germany; University of Helsinki

**Keywords:** antimicrobial resistance, processing plants, carbapenem, detection, pathogen

## Abstract

The wastewater of livestock slaughterhouses is considered a source of antimicrobial-resistant bacteria with clinical relevance and may thus be important for their dissemination into the environment. To get an overview of their occurrence and characteristics, we investigated process water (*n* = 50) from delivery and unclean areas as well as wastewater (*n* = 32) from the in-house wastewater treatment plants (WWTPs) of two German poultry slaughterhouses (slaughterhouses S1 and S2). The samples were screened for ESKAPE bacteria (*Enterococcus* spp., Staphylococcus aureus, Klebsiella pneumoniae, Acinetobacter baumannii, Pseudomonas aeruginosa, *Enterobacter* spp.) and Escherichia coli. Their antimicrobial resistance phenotypes and the presence of extended-spectrum-β-lactamase (ESBL), carbapenemase, and mobilizable colistin resistance genes were determined. Selected ESKAPE bacteria were epidemiologically classified using different molecular typing techniques. At least one of the target species was detected in 87.5% (*n* = 28/32) of the wastewater samples and 86.0% (*n* = 43/50) of the process water samples. The vast majority of the recovered isolates (94.9%, *n* = 448/472) was represented by E. coli (39.4%), the A. calcoaceticus-A. baumannii (ACB) complex (32.4%), S. aureus (12.3%), and K. pneumoniae (10.8%), which were widely distributed in the delivery and unclean areas of the individual slaughterhouses, including their wastewater effluents. *Enterobacter* spp., *Enterococcus* spp., and P. aeruginosa were less abundant and made up 5.1% of the isolates. Phenotypic and genotypic analyses revealed that the recovered isolates exhibited diverse resistance phenotypes and β-lactamase genes. In conclusion, wastewater effluents from the investigated poultry slaughterhouses exhibited clinically relevant bacteria (E. coli, methicillin-resistant S. aureus, K. pneumoniae, and species of the ACB and Enterobacter cloacae complexes) that contribute to the dissemination of clinically relevant resistances (i.e., *bla*_CTX-M_ or *bla*_SHV_ and *mcr-1*) in the environment.

**IMPORTANCE** Bacteria from livestock may be opportunistic pathogens and carriers of clinically relevant resistance genes, as many antimicrobials are used in both veterinary and human medicine. They may be released into the environment from wastewater treatment plants (WWTPs), which are influenced by wastewater from slaughterhouses, thereby endangering public health. Moreover, process water that accumulates during the slaughtering of poultry is an important reservoir for livestock-associated multidrug-resistant bacteria and may serve as a vector of transmission to occupationally exposed slaughterhouse employees. Mitigation solutions aimed at the reduction of the bacterial discharge into the production water circuit as well as interventions against their further transmission and dissemination need to be elaborated. Furthermore, the efficacy of in-house WWTPs needs to be questioned. Reliable data on the occurrence and diversity of clinically relevant bacteria within the slaughtering production chain and in the WWTP effluents in Germany will help to assess their impact on public and environmental health.

## Abstract (precis)

Bacteria from livestock may be opportunistic pathogens and carriers of clinically relevant resistance genes, as many antimicrobials are used in both veterinary and human medicine. They may be released into the environment from wastewater treatment plants (WWTPs), which are influenced by wastewater from slaughterhouses, thereby endangering public health. Moreover, process water that accumulates during the slaughtering of poultry is an important reservoir for livestock-associated multidrug-resistant bacteria and may serve as a vector of transmission to occupationally exposed slaughterhouse employees. Mitigation solutions aimed at the reduction of the bacterial discharge into the production water circuit as well as interventions against their further transmission and dissemination need to be elaborated. Furthermore, the efficacy of in-house WWTPs needs to be questioned. Reliable data on the occurrence and diversity of clinically relevant bacteria within the slaughtering production chain and in the WWTP effluents in Germany will help to assess their impact on public and environmental health.

## INTRODUCTION

Nowadays, antimicrobial-resistant bacteria involved in community and health care-associated infections give cause for serious concern for global public health. Thus, the treatment of these infections is currently one of the main challenges for humanity (1). Together with Escherichia coli, multidrug-resistant ESKAPE bacteria (*Enterococcus* spp., Staphylococcus aureus, Klebsiella pneumoniae, Acinetobacter baumannii, Pseudomonas aeruginosa, and *Enterobacter* spp.) cause the majority of life-threatening bacterial infections in health care facilities among critically ill and immunocompromised patients worldwide (2, 3). However, neither commensal E. coli bacteria nor ESKAPE bacteria are generally pathogenic (4), as most of them (i.e., E. coli, S. aureus, K. pneumoniae, *Enterobacter* spp., *Enterococcus* spp.) are natural colonizers of humans (5) and animals (i.e., livestock) (6–8). In contrast, Acinetobacter spp. and P. aeruginosa are prevalent in soil and aquatic environments, and information on their natural occurrence in animals or whether they are associated with transmission from animals to humans is scarce (9–11).

After discharge into the environment through feces and wastewater, extended-spectrum-β-lactamase (ESBL)-producing *Enterobacteriaceae* (i.e., E. coli, K. pneumoniae, *Enterobacter* spp.) and *Enterococcus* spp. may be highly prevalent in soil, plants, and surface water and may thus pose a risk for the colonization of humans (12–15), pets (16), and livestock (17, 18). Interaction with environmental pollutants (19, 20), as well as contaminated rural environments (21, 22) and food products (23, 24), can, under unfortunate circumstances, influence the composition of the microbial community of humans and animals by colonizing them with resistant ESKAPE bacteria.

One of the main properties of ESKAPE bacteria is their ability to efficiently adapt to altered environmental conditions by exchanging genetic material with other microorganisms via horizontal gene transfer (25–27). The acquisition of resistance determinants by clinically relevant bacteria can lead to an increased frequency of treatment failures and an increased severity of human infections, especially if the resistances concern antimicrobials classified as critical or highly important for human medicine (27, 28). Notable examples are the emergence of (i) extended-spectrum-β-lactamase (ESBL)-producing/fluoroquinolone-resistant *Enterobacteriaceae* (3MDRO) (29, 30) in health care settings (31) and in the poultry production chain (19, 32), (ii) carbapenem-resistant *Enterobacteriaceae* in broilers, pigs, and meat products (33, 34), as well as (iii) ESBL-producing E. coli carrying mobilizable colistin resistance (*mcr*) genes (35). The emergence of antimicrobial resistances in ESKAPE bacteria is often attributed to the inappropriate use of antibiotics in human and veterinary medicine (36), followed by successive dissemination into the environment and transfer to animals and humans. The results of various studies on related isolates of methicillin-resistant S. aureus (MRSA), vancomycin-resistant enterococci (VRE), and ESBL-producing E. coli, K. pneumoniae, and A. baumannii strains from different compartments (i.e., humans, livestock, food) (37–41) support the hypothesis that livestock are a source of clinically relevant bacteria.

Due to the high numbers of processed animals, waters from different production steps in slaughterhouses represent sources of ESKAPE bacteria. Furthermore, insufficient treatment in their in-house wastewater treatment plants (WWTPs) could provide a conduit for the discharge of clinically relevant and/or resistant bacteria into the environment and community (42–44), as recently reported for colistin-resistant, carbapenem-resistant, and extremely drug-resistant (XDR) bacteria in communal, clinical, and urban German wastewater (20, 45).

The objective of the present study was to evaluate the occurrence and diversity of ESKAPE bacteria and E. coli along different slaughtering steps in two German poultry slaughterhouses. For this purpose, samples of process waters from the washing of poultry transport trucks and vehicles, the stunning facilities, scalders, and eviscerators, as well as the wastewater effluents of the in-house wastewater treatment plants, were subjected to bacterium-specific isolation procedures. Besides species identification and antimicrobial resistance testing, the recovered bacteria were subjected to molecular epidemiological classification (phylogenetic typing and multilocus sequence typing [MLST] of E. coli and VRE, *spa* typing of MRSA) and determination of the genetic basis of the β-lactam, carbapenem, and mobilizable colistin resistance. Data were used for comparison of the content of the target bacteria in waters obtained from different slaughtering steps. Based on the results, the impact of clinically relevant bacteria released into the environment by insufficient wastewater management was assessed.

## RESULTS

### Clinically relevant resistant bacteria were detected in the majority of the investigated samples.

Within this study, 41 samples were collected from two individual slaughterhouses (slaughterhouses S1 and S2) at seven sampling points: transport trucks (only S2, *n* = 5), transport crates (*n* = 5 for each slaughterhouse), stunning facilities (*n* = 5 each), scalding water (*n* = 5 each), eviscerators (*n* = 5 each), production facilities (only S1, *n* = 5), and the influent (*n* = 8 each) and effluent (*n* = 8 each) of the in-house WWTPs. Further information on the sampling campaign dates is summarized in Table S1 in the supplemental material.

**FIG 1:**
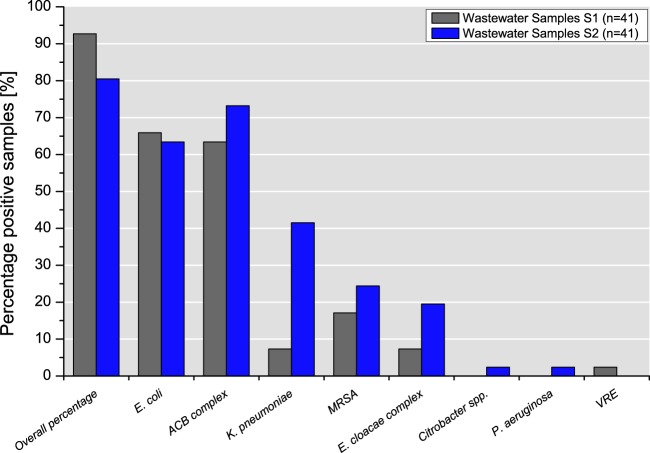
Percentage of positive samples per target bacterium in slaughterhouses S1 and S2.

Overall, 92.7% (*n* = 38) and 80.5% (*n* = 33) of the samples from S1 and S2, respectively, were positive for at least one of the ESKAPE target bacteria or ESBL-producing E. coli (Fig. 1). Detailed information about the proportion of samples positive for each species detected at each sampling point in S1 and S2 is given in Table S1.

**FIG 2:**
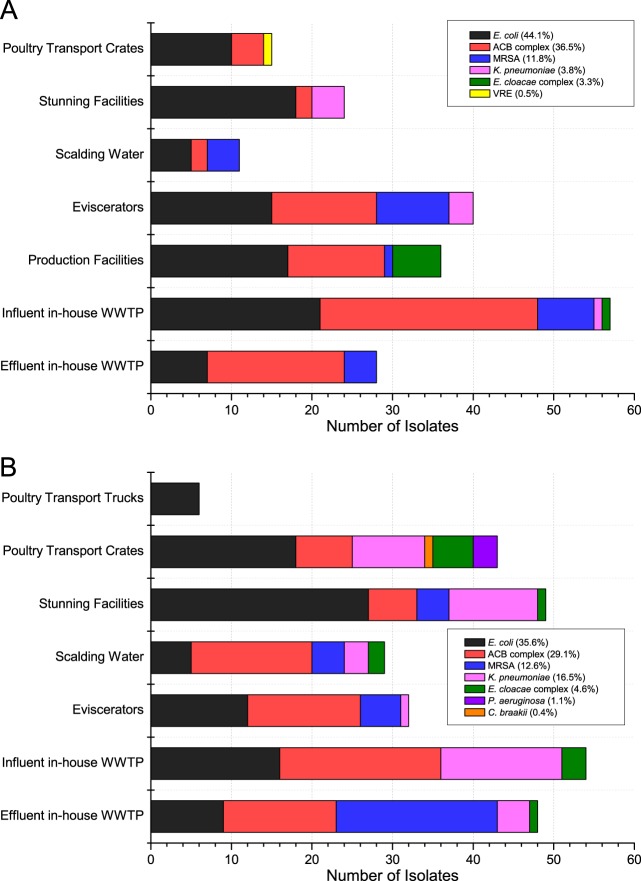
Occurrence of target bacteria across the sampling points in slaughterhouses S1 (*n* = 211) (A) and S2 (*n* = 261) (B).

Escherichia coli (65.9%, *n* = 27) and isolates of the A. calcoaceticus-A. baumannii (ACB) complex (63.4%, *n* = 26) growing on ESBL selection plates were detected in S1 at all seven sampling points (Fig. 2). VRE were detected in only one sample (2.4%), obtained from the cleaning of poultry transport crates (Fig. 2A). Interestingly, 75% of the effluent samples (*n* = 6) of the S1 WWTP were positive for the ACB complex, which grew on ESBL agar plates, whereas ESBL-producing E. coli (25.0%, *n* = 2) and MRSA (12.5%, *n* = 1) were detected less frequently (Table S1).

Similar to the findings for slaughterhouse S1, isolates of the ACB complex and E. coli were selected on ESBL plates from 73.2% (*n* = 30) and 63.4% (*n* = 26) of the samples from S2, respectively. In contrast to the findings for S1, ESBL-producing K. pneumoniae strains were detected in 41.5% (*n* = 17) of the samples from S2 at six out of seven sampling points (Fig. 2B). Furthermore, the growth of *Citrobacter* spp. and P. aeruginosa occurred sporadically in one sample (2.4% each). Effluent samples of the S2 WWTP were positive for ACB complex isolates (75.0%, *n* = 6), MRSA (62.5%, *n* = 5), E. coli (37.5%, *n* = 3), K. pneumoniae (25.0%, *n* = 2), and an isolate of the Enterobacter cloacae complex (12.5%, *n* = 1) (Table S1).

### The target bacteria exhibited a high diversity of antimicrobial resistance phenotypes.

**FIG 3:**
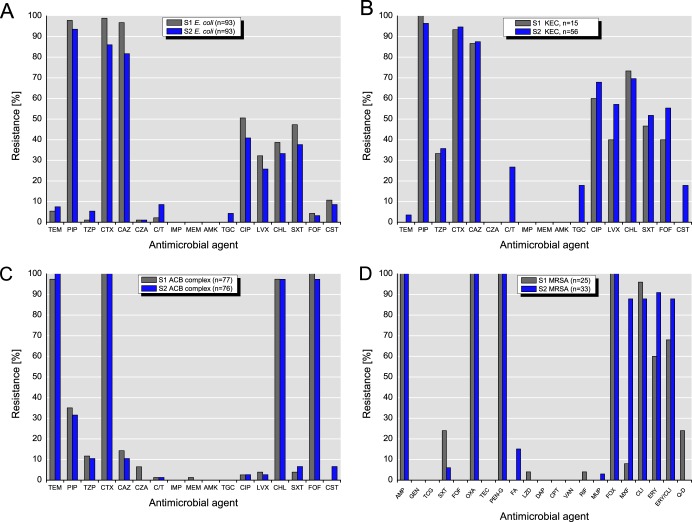
Resistance to antimicrobial agents detected among isolates of E. coli (A), K. pneumoniae, the E. cloacae complex, and *Citrobacter* spp. (B), the ACB complex (species of the ACB complex are considered intrinsically resistant to temocillin, cefotaxime, chloramphenicol, and fosfomycin) (C), and MRSA (D). Abbreviations for antimicrobial agents: TEM, temocillin; PIP, piperacillin; TZP, piperacillin-tazobactam; CTX, cefotaxime; CAZ, ceftazidime; CZA, ceftazidime-avibactam; C/T, ceftolozane-tazobactam; IMP, imipenem; MEM, meropenem; AMK, amikacin; TGC or TCG, tigecycline; CIP, ciprofloxacin; LVX, levofloxacin; CHL, chloramphenicol; SXT, sulfamethoxazole-trimethoprim; FOF, fosfomycin; CST, colistin; AMP, ampicillin; GEN, gentamicin; OXA, oxacillin; TEC, teicoplanin; PEN-G, penicillin G; FA, fusidic acid; LZD, linezolid; DAP, daptomycin; CPT, ceftaroline; VAN, vancomycin; RIF, rifampin; MUP, mupirocin; FOX, cefoxitin; MXF, moxifloxacin; CLI, clindamycin; ERY, erythromycin; Q-D, quinupristin-dalfopristin (Synercid). For temocillin with the *Enterobacteriaceae*, a breakpoint of ≤32 μg/ml for susceptible and 32 μg/ml for resistance from the British Society for Antimicrobial Chemotherapy (BSAC; 2016) was used, as there are currently no EUCAST or CLSI breakpoints.

An overview on the antimicrobial resistance of the investigated target bacteria is presented in Fig. 3. It must be kept in mind, however, that the strains were isolated from selective agar plates containing cephalosporins, oxacillin, or vancomycin; therefore, susceptibility to the selective agents cannot be expected.

Almost all E. coli isolates (*n* = 186) showed resistance to penicillins (piperacillin) and 3rd-generation cephalosporins (cefotaxime, ceftazidime). The rates of resistance to three antibiotics (i.e., piperacillin, ciprofloxacin, and 3rd-generation cephalosporins) among 3MDRO isolates were 50.5% (S1, *n* = 47/93) and 40.9% (S2, *n* = 38/93). However, only one E. coli isolate from S2 (1.1%, *n* = 1) expressed resistance to ciprofloxacin, 3rd-generation cephalosporins, and piperacillin in combination with tazobactam. While 10.8% (S1, *n* = 10) and 8.6% (S2, *n* = 8) of the isolates were resistant to colistin, all isolates were susceptible to meropenem, imipenem, and amikacin.

Similar to the findings for E. coli, almost all *Klebsiella* spp., E. cloacae complex isolates, and *Citrobacter* spp. (KEC isolates; *n* = 71) from slaughterhouses S1 (*n* = 15) and S2 (*n* = 56) showed resistance to piperacillin and 3rd-generation cephalosporins. However, the percentages of 3MDRO bacteria in S1 (60.0%, *n* = 9) and S2 (67.9%, *n* = 38) were higher than the percentages of E. coli isolates. Furthermore, none of the KEC isolates from S1 and 19.6% (*n* = 11) of the KEC isolates from 2S were resistant to ciprofloxacin, 3rd-generation cephalosporins, and piperacillin in combination with tazobactam. In contrast to the findings for S1, 17.9% (*n* = 10) and 26.8% (*n* = 15) of the KEC isolates from S2 exhibited resistance to tigecycline and ceftolozane-tazobactam, respectively. Furthermore, 17.9% (*n* = 10) of the KEC isolates from S2 were resistant to colistin.

While all isolates of the A. calcoaceticus-A. baumannii (ACB) complex (*n* = 153) from S1 and S2 were resistant to cefotaxime (these species are considered intrinsically resistant to cefotaxime, fosfomycin, chloramphenicol, and trimethoprim, according to EUCAST expert rules, v.3.1, 2016), their levels of resistance to piperacillin (S1, 35.1%; S2, 31.6%) and ceftazidime (S1, 4.3%; S2, 10.5%) were lower. The rates of 3MDRO were equally low at 2.6% (*n* = 2). Resistance to colistin was detected only in the ACB complex isolates from S2 (6.6%, *n* = 5). While all isolates were susceptible to imipenem, amikacin, and tigecycline, one isolate from S1 was meropenem resistant.

AmpC β-lactamase production was detected only in some isolates exhibiting resistance to combinations of β-lactam and β-lactamase inhibitor (S1, *n* = 24; S2, *n* = 42). Of these, 8.3% (*n* = 2) were detected in slaughterhouse S1 and 7.1% (*n* = 3) were detected in slaughterhouse S2, representing isolates of the ACB complex (*n* = 2) from S1 as well as isolates of the E. cloacae complex (*n* = 2) and a K. pneumoniae isolate (*n* = 1) from S2.

Antimicrobial resistance testing of the MRSA isolates (*n* = 58) from S1 (*n* = 25) and S2 (*n* = 33) showed that all isolates were resistant to oxacillin, ampicillin, penicillin G, and cefoxitin. The MRSA isolates from S2 showed high rates of resistance to erythromycin (90.9%) and erythromycin-clindamycin (87.9%). Furthermore, almost all MRSA isolates from S2 (87.9%, *n* = 29) were resistant to moxifloxacin, whereas the proportion of isolates from S1 with such resistance was 8.0% (*n* = 2).

The single VRE isolate from S1 was identified to be an Enterococcus faecium isolate and was determined to be resistant to oxacillin, penicillin G, gentamicin, clindamycin, daptomycin, erythromycin, vancomycin, a combination of erythromycin and clindamycin, as well as cefoxitin.

Information on the antimicrobial resistance patterns of individual isolates is given in Table S2.

### Characterization of β-lactamase genes (*bla*_ESBL_ and carbapenemase genes).

ESBL-producing E. coli (*n* = 186), K. pneumoniae (*n* = 51), E. cloacae complex (*n* = 19), and *Citrobacter* (*n* = 1) isolates were screened for the presence of *bla* genes encoding the enzymes SHV, TEM, and CTX-M.

**FIG 4:**
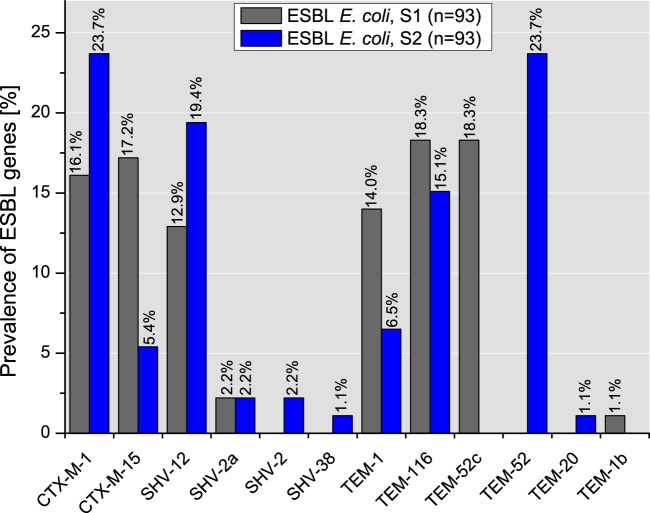
Distribution of single ESBL types in E. coli isolates from slaughterhouses S1 and S2.

Among 93 E. coli isolates from slaughterhouse S1, the most common gene was *bla*_TEM_ (51.6%, *n* = 48), followed by *bla*_CTX-M_ (33.3%, *n* = 31) and *bla*_SHV_ (15.1%, *n* = 45). Further analysis revealed the following subtypes: TEM-116, TEM-52c, and TEM-1. Among the CTX-M-positive isolates, the subtypes CTX-M-15 and CTX-M-1 were identified. *bla*_SHV_-carrying isolates exhibited the variants SHV-12 and SHV-2a (Fig. 4).

Similar to the findings for the E. coli isolates from S1, *bla*_TEM_ (46.2%, *n* = 43/93) was the most frequent gene among the E. coli isolates from S2, followed by *bla*_CTX-M_ (29.0%, *n* = 27) and *bla*_SHV_ (24.7%, *n* = 23). Further sequencing revealed that TEM-52, CTX-M-1, SHV-12, and TEM-116 were present in the vast majority of the isolates (81.5%). The minority of *bla* subtypes (18.5%) were represented by TEM-1, CTX-M-15, SHV-2, and SHV-2a, as well as SHV-38 and TEM-20 (Fig. 4).

**FIG 5:**
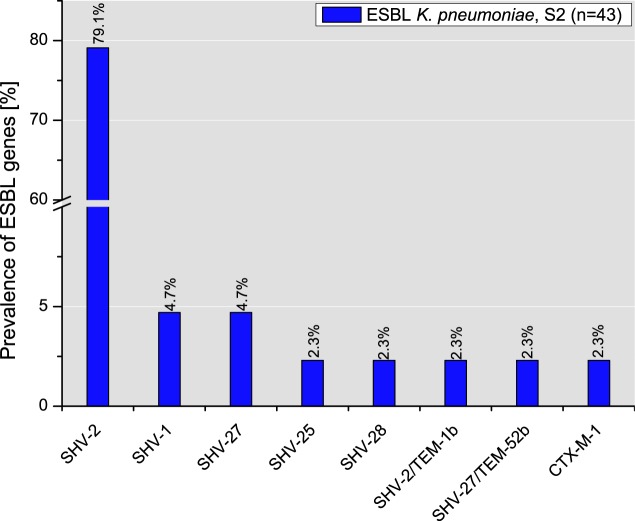
Distribution of single ESBL types in K. pneumoniae isolates from slaughterhouse S2.

The majority of the isolates of the E. cloacae complex (84.2%, *n* = 16/19) were negative for the tested ESBL genes. Only two isolates from S1 (33.3%) and one from S2 (7.7%) carried *bla*_SHV-12_. Among the *bla*_ESBL_ genes harbored by the K. pneumoniae isolates from slaughterhouse S1 (*n* = 8), SHV-2 was produced by 50.0% of the isolates, followed by SHV-28 (25.0%) and SHV-27 and SHV-1 (12.5% each). Similar to the findings for the S1 isolates, the ESBL production genotype of K. pneumoniae isolates (*n* = 43) from S2 was mainly represented by *bla*_SHV_ subtypes (97.7%). While the majority of the isolates (79.1%) expressed SHV-2, SHV-27, and SHV-1, SHV-28, SHV-25, CTX-M-1, as well as the combinations SHV-2-TEM-1b and SHV-27-TEM-52b were produced by only some K. pneumoniae isolates (Fig. 5).

*Enterobacteriaceae* isolates from CHROMagar ESBL plates (meropenem resistance cutoff, >0.125 mg/liter; *n* = 10) as well as from CHROMagar mSuperCarba plates (*n* = 22) were negative for the carbapenemase genes tested for in the molecular screening. However, we found two isolates of the ACB complex (2.6%) from S1 that tested positive for *bla*_PER_ and *bla*_GES_.

Among the isolates from S1, resistance to colistin was observed only in E. coli (10.8%, *n* = 10), while in S2, isolates of E. coli (8.6%, *n* = 8), K. pneumoniae (14.0%, *n* = 6), the E. cloacae complex (33.3%, *n* = 4), the ACB complex (6.5%, *n* = 5), and P. aeruginosa (*n* = 2) were detected. Among the colistin-resistant E. coli isolates from S1 and S2, 80% (*n* = 8) and 62.5% (*n* = 5), respectively, carried the mobilizable colistin resistance gene *mcr-1*. Besides being found in E. coli, *mcr-1* was also detected in 50% of the K. pneumoniae isolates (*n* = 3). Sanger sequencing of the *mcr-1* amplicons revealed that all analyzed isolates exhibited *mcr-1.1*.

### Phylogenetic typing and MLST of E. coli confirmed that the isolates mainly belong to the commensal bacteria.

The majority of the E. coli isolates from slaughterhouse S1 (67.7%, *n* = 63) belonged to phylogroups C (34.4%, *n* = 32) and B1 (33.3%, *n* = 31), which are mainly represented by commensal isolates. The less abundant phylogenetic groups of the E. coli isolates from S1 were phylogroups A (8.6%, *n* = 8), E (4.3%, *n* = 4), and F (3.2%, *n* = 3). The virulence-associated groups B2 and D were represented by 14.0% (*n* = 13) and 2.2% (*n* = 2) of the isolates, respectively. These isolates mainly originated from cleaning samples of the eviscerators and aggregate wastewaters from the production facilities (33.3% each, *n* = 5 each), but also from the influent of the in-house WWTP (20.0%, *n* = 3) as well as from cleaning samples from the poultry transport crates and the effluent of the in-house WWTP (13.4%, *n* = 2). The rate of resistance among 3MDRO extraintestinal pathogenic E. coli (ExPEC) isolates (26.7%, *n* = 4) was lower than the overall resistance level among 3MDRO ESBL-producing E. coli isolates from S1 (50.5%, *n* = 47). The large majority of the B2 isolates (84.6%, *n* = 11) carried *bla*_SHV-12_, whereas the remaining B2 isolates and D isolates harbored *bla*_TEM-52c_ (15.4% each, *n* = 2 each).

Similar to the findings for the E. coli isolates from S1, one of the most predominant phylogenetic groups among the S2 E. coli isolates was B1 (36.6%, *n* = 34). Phylogroups A and C exhibited equal proportions of 17.2% (*n* = 16), followed by groups E and F (14.0% each, *n* = 13 each). One isolate recovered from the effluent of the in-house WWTP belonged to group D (1.1%), and no isolates of group B2 were detected.

MLST was performed on (i) isolates belonging to phylogenetic groups B2 and D (*n* = 16), (ii) isolates recovered from the effluent of the in-house WWTPs (*n* = 12), and (iii) E. coli isolates carrying either *bla*_CTX-M-1_ or *bla*_CTX-M-15_ and expressing a 3MDRO phenotype (*n* = 34). Overall, 71.0% (*n* = 44) were assigned to 12 known sequence types (STs), and 29.0% (*n* = 18) exhibited 13 not yet assigned STs.

MLST of the E. coli isolates from phylogroups B2 and D (S1, *n* = 15) revealed that ST4994 was the most predominant sequence type among the group B2 isolates (84.6%, *n* = 11). Other individual isolates were classified as ST135 (*n* = 1) or belonged to a yet unknown sequence type (*n* = 1). Among the isolates of phylogroup D (*n* = 2, *bla*_TEM-52c_), ST69 and ST648 were detected. Isolates recovered from the effluent of the WWTPs (*n* = 6) were assigned to ST361 (*n* = 2/6, group C, *bla*_CTX-M-15_), whereas the remaining isolates (*n* = 4) exhibited yet unassigned types. Among the E. coli isolates carrying *bla*_CTX-M_ genes with the 3MDRO phenotype (*n* = 19), ST361 (78.9%, *n* = 15) was the most predominant sequence type. Four isolates could not be allocated to previously reported STs.

The isolate of phylogroup D from S2 was assigned to ST117 and expressed SHV-12. Four further isolates from the in-house WWTP effluent (*n* = 4/8) were assigned to ST10 (*n* = 2/8; group A; *bla*_TEM-52c_ and *bla*_SHV-12_, respectively), ST101 (*n* = 1/8, group B1, *bla*_CTX-M-1_) and ST212 (*n* = 1/8, group B1, *bla*_TEM-52_), whereas three isolates had unassigned STs. The E. coli isolates harboring *bla*_CTX-M_ genes and expressing the 3MDRO phenotype (*n* = 15) revealed 10 different STs. Of these isolates, 60.0% (*n* = 9) belonged to ST6617 (*n* = 3), ST1485 (*n* = 2), ST4994 (*n* = 2), and ST5686 (*n* = 2) and 40.0% (*n* = 6) of them belonged to STs that have not yet been assigned. The Escherichia coli isolates with unassigned allelic profiles are described in Table S3.

### MRSA isolates from wastewater belong to CC398 and CC9.

MRSA isolates from S1 (*n* = 25) and S2 (*n* = 33) were allocated to six different *spa* types. Five of them were livestock associated and belonged to clonal complex 9 (CC9; *spa* types t1430 and t13177) and CC398 (*spa* types t8588, t011, and t034), whereas one isolate from S1 (4.0%) was assigned to the health care-associated *spa* type t045 of CC5. It was isolated from the aggregated wastewater of the production facilities. The most predominant *spa* type among the MRSA isolates from S1 was t034 (76.0%, *n* = 19), followed by t011 (12.0%, *n* = 3) and t8588 (8.0%, *n* = 2). Of the MRSA strains from slaughterhouse S2, 75.8% (*n* = 25) belonged to *spa* type t1430, whereas 24.2% were assigned to *spa* types t034 and t13177 (12.1% each, *n* = 4 each).

### Vancomycin-resistant enterococci.

The vancomycin-resistant E. faecium isolate was allocated to ST1249 and carried the *vanA* gene.

## DISCUSSION

In this study, we investigated (i) process waters from different stages of the poultry slaughtering process as well as (ii) the influents and effluents of in-house wastewater treatment plants of two German slaughterhouses. We are not aware of previous studies in Germany in which such samples have been taken directly from the slaughterhouses and their on-site WWTPs. Our results showed that bacteria resistant to highly and critically important antimicrobials pollute the receiving water bodies, as they survived passage through the in-house WWTPs. These results clearly demonstrate that additional or alternative treatment steps are necessary before slaughterhouse wastewater can be released into the environment. The inefficacy of conventional biological WWTPs in Germany was also already reported for the treatment of municipal (45) and hospital (20) sewage.

The presence of potential clinically relevant ESKAPE bacteria (i.e., A. baumannii, K. pneumoniae) and ESBL-producing E. coli in most of the investigated stages of the poultry slaughtering process is not surprising, as most of them are able to colonize the gastrointestinal tract of livestock (especially poultry), which was also described by other authors (40, 46). Bustillo-Lecompte and Mehrvar verified that their release into the process waters and, subsequently, into the wastewater is a consequence of the excretion of organic matter from colonized poultry during delivery and slaughter (47). However, to reduce the spread of resistant bacteria into the environment, the implementation of several mitigation measures during poultry primary production (i.e., breeding farms, hatcheries, fattening farms), the slaughtering process, as well as the wastewater treatment process needs to be taken into consideration. This includes the improvement of poultry welfare conditions, the implementation of strict health and infection control programs, as well as reduction of the use of antimicrobials (48, 49).

Furthermore, during poultry processing, various intervention options, depending on the production step, are conceivable but are often not realized on the basis of their estimated cost-benefit ratios. To reduce the release of potentially dangerous bacteria into the process water used for the cleaning of poultry crates, the use of predisinfection equipment prior to the washing step is conceivable (50). The process waters applied during CO_2_ stunning and evisceration represent further important reservoirs for the dissemination of the bacteria targeted in this study, which were released by defecation, fecal leakages, and gastrointestinal disruptions. Another strategy to reduce dissemination is a general reduction of clinically relevant bacteria on the surface and in the gastrointestinal tract of the animals. This can be achieved by the inclusion of probiotics and/or prebiotics in the feed (51), the administration of oral bacteriophage cocktails (52), and the use of competitive exclusion cultures (53). Furthermore, the use of nonimmersion scalders combined with the decontamination of carcasses with hot water could decrease the level of cross-contamination with resistant bacteria (54). Besides, using moisturized hot air would decrease the amount of wastewater produced. Moreover, the use of advanced wastewater treatment technologies and wastewater disinfection needs to be considered. Hembach et al. proposed that oxidative, adsorptive, and membrane-based technologies be combined in order to prevent environmental contamination with antibiotic resistance determinants and facultative pathogenic bacteria (i.e., ESKAPE bacteria) (55).

Unlike hospital and urban effluents, where the occurrence of XDR and carbapenemase-producing bacteria is frequently described (20, 56, 57), wastewater and process water from the investigated poultry slaughterhouses did not exhibit such high-risk bacteria. This emphasizes the importance of the restricted use of carbapenems in human medicine. Moreover, the use of aminopenicillins and their β-lactamase inhibitor combinations, (fluoro)quinolones, as well as 3rd- and 4th-generation cephalosporins in livestock should be limited, and these antimicrobials should be used only to the extent that is absolutely necessary (58). They have wider spectra of action and, thus, are more likely to select for multidrug-resistant organisms, thereby compromising the activity of these antimicrobials for the treatment of severe infections in human medicine (58).

Our results on the prevailing E. coli bacteria showed a strong congruence with data published in previous papers (59, 60). Furthermore, in this study, E. coli isolates of phylogroups B2, D, and F, implicated as extraintestinal pathogens (ExPEC) (61), were recovered from all sampling locations in both slaughterhouses. This emphasizes the increased risk of transmission of such ExPEC clones to the personnel involved in particular operations in the delivery and unclean areas of the slaughtering process (62).

In general, the ESBL-producing E. coli isolates found in this study showed higher rates of resistance to fluoroquinolones than the isolates from retail chicken meat (20.8%) reported by Casella et al. (63). The increased fluoroquinolone resistance rate in the isolates of this study may be caused by the use of enrofloxacin in slaughtered broiler herds. However, reliable data on the use of fluoroquinolones in the flocks are lacking.

While the majority of the isolates could be epidemiologically linked to poultry, some of the determined sequence types/clonal lineages were also attributed to the high-risk clones (i.e., ST69, ST10, ST648 and ST117) emerging in human infections in different countries (39, 64, 65). Isolates belonging to ST69, ST10, and ST212 were detected in cleaning samples from transport crates and the effluents of the WWTPs. In general, E. coli isolates of these sequence types are high-risk clones that have been isolated from broilers and poultry meat (66), as well as from various patients with infections in different countries (65). Previously, E. coli ST212 isolates were identified to be enterotoxigenic Escherichia coli (ETEC) isolates that have been recovered from surface water, pigs, broilers, and humans (67, 68).

In this study, the majority of the ESBL-producing E. coli isolates exhibited genes that code for CTX-M-1, TEM-116, TEM-52, and SHV-12 β-lactamases. These enzymes have already been reported in isolates from poultry and humans (19, 69). Among our isolates, *bla*_CTX-M-1_ belongs to the most abundant resistance determinants in ESBL-producing E. coli, which correlates well with prevalence data for *bla*_CTX-M-1_ (18.0 to 69.0%) in isolates from chickens and chicken meat in Germany (70, 71). Similar to the findings of other studies, E. coli isolates carrying *bla*_SHV-2_, *bla*_SHV-2a_, and *bla*_TEM-20_ were only sporadically detected in chicken and retail chicken meat (72, 73). In contrast to the findings of other studies from Germany and the Netherlands (70, 74, 75), where low percentages (0.0 to 5.2%) of *bla*_CTX-M-15_-producing E. coli isolates in chickens and poultry products have been identified, 12.2% of the ESBL-producing E. coli isolates from the wastewater of slaughterhouse S1 carried this gene. This may have been due to the possible acquisition of *bla*_CTX-M-15_ plasmids from human strains, as has already been shown for animal E. coli strains in the United Kingdom (76). CTX-M-15 is one of the most frequently encountered ESBL enzymes in human clinical isolates from various countries (77). This is partially due to the clonal spread and predominance of a subset of ExPEC lineages in the human population that are commonly associated with *bla*_CTX-M_ genes (particularly with *bla*_CTX-M-15_), e.g., ST131, ST69, and ST10 (39, 78). However, the abundance of such ExPEC clones in poultry production in Germany is moderate, and often they are associated with pAmpC rather than *bla*_CTX-M_ (59). In this context, the transfer of *bla*_CTX-M-15_ in E. coli by mobile genetic elements between humans, livestock, and the environment through the food chain (79) and occupational exposure (80) may play a primary role.

In previous studies, ESBL-producing K. pneumoniae isolates were only sporadically identified in German broilers during slaughter (81, 82), whereas in this study, 63.4% of the samples in almost all sampling locations in slaughterhouse S2 tested positive for K. pneumoniae. In contrast to samples from S2, only 7.3% of the samples from S1 exhibited K. pneumoniae. These different proportions might be caused by the content of the colonized flocks that were supplied by different fattening farms. Taking into consideration that there are only a few breeding companies and hatcheries in Germany, the possibility of vertical transmission through the production chain cannot be excluded. Moreover, the majority of the K. pneumoniae isolates carried *bla*_SHV-2_, which has also been frequently found in isolates from egg shells, broilers, and humans (46, 83). The results of our study are consistent with the observations that resistance genes of the SHV β-lactamase family are ubiquitous in ESBL-producing K. pneumoniae strains (84, 85). However, in contrast to the isolates occurring in environmental sources in rural areas (86) as well as those cultured from patient specimens (87), almost all K. pneumoniae isolates from this study lacked genes of the CTX-M/TEM families. This emphasizes the need for further studies applying high-resolution technologies, such as whole-genome sequencing, to better elucidate their epidemiology and clinical relevance.

To date, only limited data on *Enterobacter* spp. exhibiting resistance to 3rd- and 4th-generation cephalosporins in poultry are available. Overall, the occurrence of bacteria of the E. cloacae complex (1.23%) in retail poultry meat seems to be low, as previously reported for Germany (72). ESBL-producing bacteria of the E. cloacae complex (15.8%) exhibited only the ESBL enzyme SHV-12. These data are in good agreement with the observations of Towne et al. (88), who detected *bla*_SHV-12_ in 8.7% of clinical *Enterobacter* isolates. A steadily increasing abundance of ESBL-encoding isolates of the E. cloacae complex along with the inducible production of AmpC, as well as its constitutive derepression, could lead to nearly pan-resistance to β-lactam antibiotics, diminishing the already limited number of therapeutic options (89–91).

Bacteria of the ACB complex belong to the most important nosocomial pathogens and are able to survive in competitive and demanding environments (92). However, reliable data on the impact of livestock-associated isolates on human and animal health are lacking. Wilharm et al. indicated linkages between livestock isolates and human clinical isolates, suggesting that A. baumannii might have zoonotic potential (40). Despite the importance of these bacteria, data on the emergence and antimicrobial resistance of bacteria of the ACB complex in broilers are scarce (40, 93). However, among the raw meats tested, poultry meat retailed in different countries showed the highest rates of contamination with species of the ACB complex (26.7% to 48.0%) (94).

As bacteria of the ACB complex were sporadically isolated from chickens, goslings, ducks, and wild birds (40, 95), these animals might play an important role as natural reservoirs for these organisms. Given that Acinetobacter spp. are considered intrinsically resistant to cefotaxime, temocillin, fosfomycin, and chloramphenicol (96), the ACB complex isolates in our study contrast with human and animal clinical strains, which exhibited high levels of resistance to fluoroquinolones and carbapenems (97, 98). Almost all tested isolates lacked acquired *bla*_ESBL_ genes, like *bla*_CTX-M_, *bla*_TEM_, *bla*_SHV_, *bla*_VEB_, *bla*_PER_, and *bla*_GES_, which would be genetic evidence of acquired resistance to β-lactams (92). However, their resistance to 3rd-generation cephalosporins might be a consequence of the increased expression of the chromosomal *bla*_ADC_ gene or other mechanisms (99).

The results of this study show that the majority of the detected MRSA lineages belonged to CC398 and ST9, which are the most common livestock-associated MRSA (LA-MRSA) lineages in Europe (100). *spa* types t034, t011, and t1430 have already been identified among isolates from chicken and meat products in countries with pronounced conventional farming, like Denmark (101), Germany, and the Netherlands (102). Furthermore, they were also detected in environmental samples from broiler barns (14), poultry slaughterhouses and their personnel (103), as well as human patients in Norway and different countries of the European Union/European Economic Area (38, 104). Interestingly, isolates of *spa* types t034 and t011 represent the most frequent LA-MRSA isolates recovered from hospital inpatients and ambulatory patients in Germany in regions with high levels of livestock production (38). Another notable finding was the detection of *spa* type t13177 in MRSA isolates from the effluent of the WWTPs. Isolates of this type were sporadically detected in fresh broiler meat and retail chicken meat in Germany (105) and Switzerland (106), respectively. Unlike the other LA-MRSA isolates, these isolates carried genes coding for major staphylococcal enterotoxins, which may cause toxic shock-like syndromes and which are implicated in food poisoning (107).

The antibiotic resistance patterns of the MRSA isolates in this study are similar to those of the isolates described by Rosenberg Goldstein et al. (12). However, the observed differences between isolates from slaughterhouses S1 and S2 may be due to the distinct prevalent clonal lineages detected in the two slaughterhouses. In another study (103), 95.0% of t1430 MRSA isolates but only 11.5% of t034 MRSA isolates were resistant to ciprofloxacin, while these were the most predominant *spa* types in MRSA isolates from S1 and S2. It has been postulated that t1430 is a poultry-associated MRSA type (103) and that its high levels of resistance to moxifloxacin might be due to the usage of (fluoro)quinolones in the poultry industry.

Within this study, only one vancomycin-resistant E. faecium ST1249 isolate was recovered from cleaning samples from poultry transport crates. VRE ST1249 has been previously isolated from 3.7% of chicken products in the United Kingdom (108). The occurrence of VRE in livestock is related to the glycopeptide avoparcin (109, 110), which was used for growth promotion in Germany between 1975 and 1996 (111). However, Johnsen et al. (112) and Andersson and Hughes (113) suggest that the reversibility of acquired glycopeptide resistance is slow and could last for >25 years. Our findings and other reports (114, 115) reinforce this theory. Furthermore, it is presumed that without the selective pressure of avoparcin, coselection by macrolides, which are often used to treat poultry, can occur (116). Moreover, the copper added to the feed can also exert a selective effect on VRE (117).

### Conclusion.

Process water and wastewater from poultry slaughterhouses are important reservoirs for antimicrobial-resistant bacteria with clinical relevance. The ubiquitous distribution of enterobacteria and MRSA with resistances to highly and critically important antimicrobials in the process water and wastewater of poultry slaughterhouses is worrisome, as they (i) may colonize slaughterhouse workers and (ii) may be reintroduced into the food chain by cross-contamination during carcass processing. (iii) Furthermore, they are released into the environment via surface waters due to insufficient treatment within in-house WWTPs. The implementation of new measures to reduce the input of resistant bacteria into the slaughterhouses and their consequent excretion into process water and wastewater, as well as strategies for improvement of discharge water status and treatment processes, needs to be taken into consideration.

## MATERIALS AND METHODS

### Sampling and sample preparation.

Two German poultry slaughterhouses (slaughterhouses S1 and S2) exhibiting different slaughtering capacities above 100,000 chickens per day were investigated. S1 and S2 produce daily 600 m^3^ and 3,600 m^3^ wastewater, respectively. S1 operates a wastewater treatment plant (WWTP) based on the membrane bioreactor (MBR) technology with immersed ultrafiltration membranes. S2 possesses a conventional biological WWTP. After treatment, effluents are discharged into the preflooder and further into surface water bodies.

The collected samples represent various waters that accumulate during delivery, in the unclean area of the poultry slaughtering process, as well as in their in-house WWTPs. Samples were taken at seven sampling sites: transport trucks (only S2), transport crates, stunning facilities, scalders, eviscerators, production facilities (only S1), and the influent and the effluent of the in-house WWTPs. Sampling of both slaughterhouses was conducted during five independent visits between December 2016 and September 2018. Three further visits were conducted to obtain additional samples from the in-house WWTPs of S1 and S2 in the same time period. A minimum time interval of 1 month was kept between two independent sampling visits to minimize the possible carryover of the targeted bacteria from poultry flocks originating from the same fattening farm and to ensure that the individual samplings would be representative of different poultry populations.

From each individual sample, 1 liter was collected using sterile Nalgene wide-mouth environmental sample bottles (Thermo Fisher Scientific, Waltham, MA, USA). Composite samples from the precleaning of five poultry trucks after unloading of birds (only S2) and samples of water applied for the precleaning of the stunning facilities were collected by catching runoffs. In general, all precleaning steps were conducted without using cleaning or disinfection agents. Water samples from the cleaning of the poultry transport crates and scalding water were taken by immersion of sterile sampling bottles into the sump of the crate washing facility and the scalder tank, respectively. Process water used during evisceration was collected as runoffs from eviscerators in operation. Aggregate wastewater from production facilities (only S1) was taken by immersion of sterile sampling bottles into mixing and homogenization containers after the wastewater had run through a mechanical deposition. The samples of influent and effluent of the in-house WWTPs were taken as qualified samples according to the German standard methods for the examination of water, wastewater, and sludge (DIN 38402-11:2009-02) (118). The samples were labeled and transported to the laboratory in a Styrofoam box cooled to 5 ± 2°C. To get rid of coarse particles (e.g., bedding, feathers), the samples were manually filtered using stomacher strainer bags with a tissue filter (pore size, 0.5 mm; VWR, Radnor, PA, USA) and afterwards subjected to cultivation within 24 h after sampling.

### Cultivation and identification of antimicrobial-resistant target bacteria.

Water samples were screened for Gram-negative ESBL-producing bacteria of the *Enterobacteriaceae*, nonfermenting A. baumannii and P. aeruginosa, as well as methicillin-resistant S. aureus (MRSA), vancomycin-resistant enterococci (VRE), and carbapenemase-producing *Enterobacteriaceae* (CPE). To detect ESBL-producing target bacteria and VRE, 100-μl aliquots of serial 10-fold dilutions or 1 ml of undiluted samples was applied to CHROMagar ESBL and CHROMagar VRE plates (Mast Diagnostica, Reinfeld, Germany) for cultivation. Furthermore, 10- and 100-ml aliquots of the in-house WWTP effluent were filtered through sterile 0.45-μm-pore-size, 47-mm mixed cellulose nitrate filters (GE Healthcare, Chicago, IL, USA) and placed on selective agar plates. To inhibit the growth of accompanying bacteria, all agar plates were incubated at 42°C for 18 to 24 h (ESBL-producing target bacteria) and for 42 to 48 h (VRE).

MRSA isolates were recovered following the recommendations of the National Reference Laboratory for Staphylococci, with some modifications. For this purpose, 100 ml of the water samples was (i) filtered through sterile 0.45-μm-pore-size, 47-mm mixed cellulose nitrate filters (only for the effluents of the in-house WWTPs) or (ii) centrifuged for 15 min at 5,000 × *g* and 4°C. The filters or resulting pellets were transferred to 100 ml of Mueller-Hinton broth (MHB; Sigma-Aldrich, St. Louis, MO, USA) supplemented with 6.5% NaCl for preenrichment. After incubation at 37°C for 18 to 24 h under shaking (150 rpm), 1 ml of the preenrichment broth was transferred to 9 ml of tryptic soy broth (TSB; Sigma-Aldrich, St. Louis, MO, USA) supplemented with aztreonam (50 mg/liter) and cefoxitin (3.5 mg/liter). The inoculated selective preenrichment broth was cultivated for 18 to 24 h at 37°C. Afterwards, a 10-μl loopful of culture was streaked out on CHROMagar MRSA (Mast Diagnostica, Reinfeld, Germany) screening agar and incubated at 42°C for 18 to 24 h.

For the isolation of CPE, a selective preenrichment was carried out. Therefore, 10-ml water samples were subjected to filtration through 0.45-μm-pore-size membrane filters. The filters were incubated at 42°C for 18 to 24 h in Mossel broth (Sigma-Aldrich, St. Louis, MO, USA) under aerobic conditions to inhibit the growth of accompanying flora (i.e., Gram-positive microorganisms). Thereafter, 100 μl of the selective preenrichment broth was plated on CHROMagar mSuperCarba (Mast Diagnostica, Reinfeld, Germany) plates and incubated at 42°C for 18 to 24 h.

Whenever possible, up to four presumptive colonies per sampling site of E. coli, *Klebsiella* spp., *Enterobacter* spp., *Citrobacter* spp., Acinetobacter spp., *Pseudomonas* spp., MRSA, as well as VRE were picked from the selective plates and subcultured on Columbia agar with 5% sheep blood (Mast Diagnostica, Reinfeld, Germany) at 37°C for 18 to 24 h. Presumptive coliform bacteria were confirmed by streaking on Chromocult coliform agar (Merck, Darmstadt, Germany) and oxidase testing. The nonfermenting Acinetobacter spp. and *Pseudomonas* spp. were subcultured on CHROMagar Acinetobacter agar (Mast Diagnostica, Reinfeld, Germany) and cetrimide agar (Merck, Darmstadt, Germany), respectively, and confirmed by oxidase testing. Potential VRE colonies were streaked onto Slanetz Bartley agar (Merck, Darmstadt, Germany).

Species identification was conducted using matrix-assisted laser desorption ionization–time of flight mass spectrometry, employing a Vitek MS mass spectrometer (bioMérieux, Marcy l’Etoile, France) equipped with Myla software. All isolates were purified on Columbia agar with 5% sheep blood and preserved in cryotubes (Mast Diagnostics, Reinfeld, Germany) at −70°C.

### Antimicrobial susceptibility testing.

The activities of 17 antimicrobials or antimicrobial combinations against Gram-negative bacteria were tested by the microdilution method according to the protocols of the European Committee on Antimicrobial Susceptibility Testing (EUCAST) using a Micronaut-S MDR MRGN screening system (Merlin, Gesellschaft für mikrobiologische Diagnostika GmbH, Bornheim-Hersel, Germany). Resistance testing of Gram-positive bacteria was conducted using the Micronaut-S MRSA/GP testing panel. The results were interpreted according to clinical cutoff values (EUCAST, v.9.0) in order to determine the profiles of resistance of ESKAPE bacteria of livestock origin to antimicrobials medically important for humans and to assess their clinical relevance in human medicine. The 3MDRO classification of the isolates was done according to the recommendations of the Commission for Hospital Hygiene and Infection Control of 2012 (KRINKO) at the Robert Koch Institute, Berlin, Germany; i.e., intermediate was interpreted as resistant (29).

Isolates resistant to β-lactam–β-lactamase inhibitor combinations were further screened for AmpC enzyme production using the AmpC test D69C (Mast Diagnostica, Reinfeld, Germany).

### Molecular detection and typing.

For molecular analyses, the template DNA for PCR experiments was prepared from bacterial suspensions in 10 mM Tris-EDTA, pH 8.0 (Sigma-Aldrich, St. Louis, MO, USA), according to the Tris-EDTA boiling lysate method (119). ESBL-producing coliforms were screened for the presence of β-lactamase genes belonging to the *bla*_TEM_, *bla*_SHV_, and *bla*_CTX-M_ families. To this end, detection assays were used as previously described (120–122). For subtyping, *bla*_CTX-M_-positive samples were further investigated as previously described (123). Sanger sequencing was performed at Microsynth Seqlab (Göttingen, Germany) using PCR amplicons purified with an innuPREP DOUBLEpure kit (Analytik Jena AG, Jena, Germany). Sequence analysis was conducted with the Chromas lite (v.2.6.5; Technelysium Pty Ltd), BioEdit (v.7.2.5) (124), and NCBI BLAST (http://blast.ncbi.nlm.nih.gov/) programs.

According to the recommendations of EUCAST (125), isolates of *Enterobacteriaceae* picked from the CHROMagar ESBL plates (meropenem resistance cutoff, >0.125 mg/liter) as well as from the CHROMagar mSuperCarba plates were screened for the presence of the carbapenemase genes *bla*_NDM_, *bla*_IMI_, *bla*_VIM_, *bla*_OXA-48_, *bla*_KPC_, and *bla*_GES_ by multiplex real-time TaqMan PCR assays (126, 127). Acinetobacter isolates were investigated for the presence of *bla*_PER_, *bla*_GES_, and *bla*_VEB_ by PCR and sequencing (128).

The detection of *mcr* genes among colistin-resistant isolates (MICs > 2 mg/liter) was conducted by conventional PCR (129). As PCR controls, the isolates E. coli R2749 (*mcr-1*), E. coli KP37 (*mcr-2*), Salmonella enterica serovar Typhimurium SSI_AA940 (*mcr-3*), *S*. Typhimurium R3445 (*mcr-4*), and E. coli 10E01066 (*mcr-5*) were used. Sequence-based typing of the *mcr-1* amplicons was performed as previously described (130).

Determination of phylogenetic groups (phylogenetic groups A, B1, B2, C, D, E, and F and clade I) of E. coli and MLST of selected isolates (i.e., extraintestinal pathogenic E. coli [ExPEC], 3MDRO carrying *bla*_CTX-M_) was conducted using the methods of Clermont et al. (131) and Wirth et al. (132), respectively. Sequence-based MLST typing was performed using the EnteroBase database (http://enterobase.warwick.ac.uk).

For *spa* typing of the MRSA isolates, the *Staphylococcus* protein A repeat region was amplified and sequenced as previously described (133). *spa* types were predicted using SpaServer Ridom software (http://www.spaserver.ridom.de). Vancomycin-resistant enterococci were screened for the *vanA*, *vanB*, *vanC1*, and *vanC2* genes by a multiplex PCR assay as previously described (134) and MLST typed using the method of Homan et al. (135). Sequence data were analyzed using the PubMLST database (https://pubmlst.org/efaecium).

## Supplementary Material

Supplemental file 1

Supplemental file 2
