# Supplementary material for: ESKAPE Bacteria and Extended-Spectrum-β-Lactamase-Producing Escherichia coli Isolated from Wastewater and Process Water from German Poultry Slaughterhouses
Source: Appl Environ Microbiol. 2020 Apr 1;86(8):e02748-19. doi: 10.1128/AEM.02748-19 (PMC7117925; doi:10.1128/AEM.02748-19)
Supplement: Supplemental file 1 [file AEM.02748-19-s0001.pdf]

Supplementary Material

Slaughterhouses are important reservoirs for bacteria of clinical relevance: Insights into the isolation and characterization of ESKAPE-bacteria and ESBL-producing *E. coli* from waste- and process water of two German poultry slaughterhouses

Mykhailo Savin, Gabriele Bierbaum, Jens Andre Hammerl, Céline Heinemann, Marijo Parcina, Esther Sib, Alexander Voigt, Judith Kreyenschmidt

#Corresponding author:

Mykhailo Savin, Institute of Animal Sciences, University of Bonn, Katzenburgweg 7-9, D-53115 Bonn, Germany. E-mail: m.savin@uni-bonn.de

Supplement material Table S1: Number of positive samples per target bacteria and sampling point. Numbers of positive samples and total numbers of samples at each sampling point are stated.

	E. coli	ACB complex	MRSA	*K. pneumoniae*	*E. cloacae* complex	*Citrobacter spp.*	*P. aeruginosa*	VRE
				**Slaughterhouse S1[a]**				
**Poultry Transport Crates**	3/5	2/5	0/5	0/5	0/5	0/5	0/5	1/5
**Stunning Facilities**	5/5	1/8	0/5	1/5	0/5	0/5	0/5	0/5
**Scalding water**	1/5	1/5	1/5	0/5	0/5	0/5	0/5	0/5
**Eviscerators**	4/5	5/5	2/5	1/5	0/5	0/5	0/5	0/5
**Production Facilities**	5/5	4/5	1/5	0/5	2/5	0/5	0/5	0/5
**Influent in-house[b] WWTP**	7/8	7/8	2/8	1/8	1/8	0/8	0/8	0/8
**Effluent in-house[b] WWTP**	2/8	6/8	1/8	0/8	0/8	0/8	0/8	0/8
				**Slaughterhouse S2[c]**				
**Poultry Transport Trucks**	2/5	0/5	0/5	0/5	0/5	0/5	0/5	0/5
**Poultry Transport Crates**	4/5	4/5	0/5	3/5	1/5	2/5	1/5	0/5
**Stunning Facilities**	5/5	3/5	2/5	4/5	1/5	0/5	0/5	0/5
**Scalding water**	2/5	5/5	1/5	1/5	2/5	0/5	0/5	0/5
**Eviscerators**	4/5	5/5	2/5	1/5	0/5	0/5	0/5	0/5
**Influent in-house[d] WWTP**	6/8	7/8	0/5	6/8	2/8	0/5	0/5	0/5
**Effluent in-house[d] WWTP**	3/8	6/8	5/8	2/8	1/8	0/5	0/5	0/5

a – sampling campaigns in December 2016, July 2017, August 2017, December 2017, February 2018
b – additional sampling campaigns in the S1 in-house WWTP in September 2017, November 2017, March 2018
c – sampling campaigns in October 2017, November 2017, March 2018, April 2018, Mai 2018
d – additional sampling campaigns in the S2 in-house WWTP in July 2018, September 2018, October 2018

Supplement material TABLE S3: Results of the MLST analysis for *E. coli* strains with new STs[a].

Isolat ID	Sampling point	Phylogenetic group	*bla*-genes	Resistance profile[b]	Allelic profile							Nearest matches
					adk	*fumC*	*gyrB*	*icd*	*mdh*	*purA*	*recA*	
				**Slaughterhouse S1**								
**LWGS-1/5-62**	Influent in-house WWTP	B1	$bla_{\text{CTX-M-1}}$	PIP, CTX, CAZ, CIP		4	33	16	24	8	14	ST5686
**LWGS-1/7-32**	Wastewater from Stunning Facilities	B1	$bla_{\text{CTX-M-1}}$	PIP, CTX, CAZ, CIP, LVX, CHL, SXT	429	4	375	16	11	8	6	ST224, ST906, ST2186
**LWGS-1/5-71**	Influent in-house WWTP	B1	$bla_{\text{CTX-M-1}}$	PIP, CTX, CAZ, CIP, CHL	429	4	375	8	24	8	14	ST889, ST892, ST3995
**LWGS-1/6-10**	Effluent in-house WWTP	B1	$bla_{\text{CTX-M-1}}$	PIP, CTX, CAZ, CIP	429	4	375	16	24	8	14	ST3995, ST5686
**LWGS-1/6-03**	Effluent in-house WWTP	B1	$bla_{\text{CTX-M-1}}$	PIP, CTX, CAZ	429	4	375	16	24	8	14	ST3995, ST5686
**LWGS-1/6-21**	Effluent in-house WWTP	B1	$bla_{\text{CTX-M-1}}$	PIP, CTX, CAZ	429	4	375	16	24	8	14	ST3995, ST5686
**LWGS-1/3-04**	Scalding water	B1	$bla_{\text{CTX-M-1}}$	PIP, CTX, CAZ, CIP	429	4	375	16	24	8	14	ST3995, ST5686
**LWGS-1/8-05**	Aggregate Wastewater from Production Facilities	B2	$bla_{\text{TEM-52c}}$	PIP, CTX, CAZ	429	31	5	28	1	1	2	ST57, ST5860, ST8144
**LWGS-1/6-22**	Effluent in-house WWTP	B1	$bla_{\text{TEM-52c}}$	PIP, CTX, CAZ	457	6	15	131	24	7	7	ST711
				**Slaughterhouse S2**								
**LWGS-4/7-22**	Wastewater from Stunning Facilities	B1	$bla_{\text{CTX-M-1}}$	TEM, PIP, CTX, CAZ, C/T, CIP, LVX, CHL, SXT	429	29	33	16	11	7	2	ST1844
**LWGS-4/6-40**	Effluent in-house WWTP	B1	$bla_{\text{SHV-12}}$	PIP, CTX, CAZ, CHL, SXT	429	556	5	18	11	8	6	ST4663
**LWGS-4/6-41**	Effluent in-house WWTP	B1	$bla_{\text{SHV-12}}$	PIP, CTX, CAZ, CHL	429	556	5	18	11	8	6	ST4663
**LWGS-4/1-06**	Wastewater from Poultry Transport Trucks	E	$bla_{\text{CTX-M-1}}$	PIP, CTX, CAZ, CIP, LVX, SXT	52	116	55	16	113	31	38	ST4994
**LWGS-4/2-17**	Wastewater from Poultry Transport Cages	B1	$bla_{\text{CTX-M-1}}$	PIP, CTX, CAZ, CIP, LVX, CHL, SXT, CST	429	19	4	16	9	38	6	ST5203
**LWGS-4/7-23**	Wastewater from Stunning Facilities	B1	$bla_{\text{CTX-M-15}}$	PIP, CTX, CAZ, CIP, LVX	457	19	32	1	9	8	6	ST1723, ST3365
**LWGS-4/2-16**	Wastewater from Poultry Transport Cages	B1	$bla_{\text{CTX-M-1}}$	PIP, CTX, CAZ, CIP, LVX, CHL, SXT, CST	429	4	4	18	24	8	14	ST223, ST465, ST2120
**LWGS-4/2-20**	Wastewater from Poultry Transport Cages	B1	$bla_{\text{CTX-M-1}}$	PIP, CTX, CAZ, CIP, LVX, SXT	429	4	14	16	358	8	14	ST5686, ST7329
**LWGS-4/6-18**	Effluent in-house WWTP	E	$bla_{\text{CTX-M-1}}$	PIP, CTX, CAZ	429	31	5	28	1	1	2	ST57, ST5860, ST8144

[a]The ST was not assigned numerical designations by the *E. coli* MLST database (http://mlst.warwick.ac.uk/mlst/dbs/Ecoli).

[b]Abbreviations for antimicrobial agents: TEM, temocillin; PIP, piperacillin; CTX, cefotaxime; CAZ, ceftazidime; C/T, ceftolozane-tazobactam; CIP, ciprofloxacin; LVX, levofloxacin; CHL, chloramphenicol; SXT, sulfamethoxazole-trimethoprim; CST, colistin
